# Determination of cinnamaldehyde, thymol and eugenol in essential oils by LC–MS/MS and antibacterial activity of them against bacteria

**DOI:** 10.1038/s41598-024-63114-8

**Published:** 2024-05-30

**Authors:** Zhi Li, Yan Li, Wenbo Cheng

**Affiliations:** 1 Tianjin Guoke Medical Technology Development Co., LTD, Tianjin, 300399 China; 2 Suzhou Institute of Biomedical Engineering and Technology, Chinese Academy of Sciences, Suzhou, 215163 China

**Keywords:** HPLC–MS/MS, Synergistic antimicrobial, Trace metal, Essential oils, Biochemistry, Immunology

## Abstract

Plant essential oils contain many secondary metabolites, some of which can effectively inhibit the growth of pathogenic microorganisms, so it is a very promising antibacterial agent. In this study, a qualitative and quantitative method based on high performance liquid chromatography–tandem mass spectrometry (HPLC–MS/MS) was developed for the simultaneous determination of three bioactive substances, cinnamaldehyde (CNM), thymol (THY), and eugenol (EUG), in the essential oils of plants. Necessary tests for linearity, limit of quantification, recovery, carryover contamination and precision of the method were carried out. Then, the antibacterial activity of 3 bioactive compounds against *Escherichia coli* (*E. coli*) and *Staphylococcus aureus* (*S. aureus*) was evaluated by minimal inhibitory concentration and the synergistic antimicrobial effect. The results indicated that CNM, THY and EUG had good antibacterial activity. According to the results of fractional inhibitory concentration index (FICI), it is considered that CNM + THY and CNM + THY + EUG has obvious synergistic inhibitory effect on *E. coli*, and CNM + THY and CNM + EUG has obvious synergistic inhibitory effect on *S. aureus*. Finally, we analyzed the effect of the bioactive compounds on trace elements in bacteria and found significant changes in magnesium, calcium, copper and iron.

## Introduction

*Staphylococcus aureus* (*S. aureus*) is a common foodborne curing microorganism, *S. aureus* has contributed to a severe threat, involving mild skin infection, severe tissue infection, and sepsis to human health due to its widely distribution in human skin, especially in the nasopharynx. In addition, *S. aureus* can also produce enterotoxins that can cause food poisoning^1^. *Escherichia coli* (*E. coli*) is a gram-negative bacterium and a conditional pathogen. However, certain strains of *E. coli* are capable of producing toxins and have a low infectious dose, making them highly transmissible through various media. *E. coli* can survive in the environment and proliferate in food, thus posing a threat to human health^2^. Under certain conditions, *E. coli* can cause gastrointestinal tract infections, urinary tract infections and other local tissue and organ infections^3^. At present, the treatment of bacterial infections still relies heavily on antibiotics, such as chloramphenicol and tetracycline. However, the misuse of antibiotics leads to bacterial resistance and reduces the clinical effectiveness of antibiotics. Therefore, there is an urgent need to find a highly effective, low-toxicity, non-resistant product to treat bacterial infections, and plant essential oils are one of the most highly regarded natural products^1^.

Plant essential oils (EOs) is a substance originally derived from plants, also known as aromatic oil or volatile oil^4^. It contains a variety of plant secondary metabolites, the main components of plant essential oils are monoterpenes and sesquiterpenes, as well as carbohydrates, phenols, alcohols, ethers, aldehydes, and ketones, which are responsible for the bioactivity of aromatic and medicinal plants and give off their scent. Plant essential oils have a variety of pharmacological effects, such as antioxidant action, analgesic, antipyretic, hypoglycemic, antiulcer, anticonvulsant, antipathic aging and anticancer, as well as inhibiting or slowing down the growth of microorganisms, which makes them a very promising bacteriostatic agent^5^. Many essential oils and their volatile components have been found to have strong antibacterial activity, which inhibit the growth of some bacteria, fungi, and other microorganisms, and suppress the production or accumulation of mycotoxins. Wang et al. found that ginger essential oil could have good antibacterial effects on the *S. aureus* and *E. coli*^3^. The antibacterial activity of essential oils is mainly caused by its complex active components (mainly including alcohols, aldehydes, ketones, and phenols). Essential oils can use its own hydrophobicity to penetrate lipids, thus destroying the structure of the cell wall and changing the permeability of the cell membrane: this leads to the outflow of ions and matter within the cyst, causing cell death. And it has been found that combining two or more compounds can be more effective in enhancing antioxidant and antimicrobial activities and has synergistic bacteriostatic potential^6^. The use of plant EOs as antibacterial agents not only implies greater safety for humans and a more environmentally friendly option due to their natural origin, but also represents a low risk of pathogenic microorganisms developing resistance^7^. However, the use of phytochemicals to prevent foodborne microbes is poorly studied. In this study, a novel LC–MS/MS method was constructed to determine bioactive compounds levels in chili essential oils. Following these results, this work was designed to validate a highly sensitive and selective method for the qualitative and quantitative determination of these components in the chili essential oils (CEOs). The purpose of this study was to extend the functional potential of CEOs by investigating their antibacterial activity against two major food-borne bacteria and also to examine the possible underlying antibacterial mechanisms.

## Materials and methods

### Chemicals and reagents

All solvents used in the current experiment were of HPLC grade. All chemicals and reference powders were of AR grade. Cinnamaldehyde (CNM), cinnamic acid (CA), thymol (THY) and eugenol (EUG) reference powder were procured from Solarbio (Beijing, China). Formic acid, methanol and acetonitrile were purchased from Sigma-Aldrich Company (St. Louis, MO, USA). In-house Milli-Q Plus purification equipment, purchased from Millipore Company (Billerica, MA, USA), generates the required water at HPLC grade (resistivity 18.2 MΩ cm)^8^.

### Microorganisms and essential oil

The Gram-positive strain: *S. aureus* and Gram-negative strains: *E. coli* were procured from our laboratory depository. These two bacteria were revived in the Luria Broth medium (Beijing Land Bridge Technology Co., Ltd., Beijing, China) at 27 °C for 16 h for activation of the test cultures. Chili essential oil was purchased from Jiangxi Jinyuan Natural Flavor Co., Ltd. (Jiangxi, China)^3^.

### Extraction of essential oils

In total, 1 g chili essential oil were added to 10 mL volumetric bottle and fill with acetonitrile to 10 mL, then CNM, THY and EUG were extracted by ultrasonic-assisted extraction (UAE) method. The extract was fully sonicated for 5 min using a sonicator (Jining, China) and centrifuged at 10,000 g. The extracts were filtered through a filter paper after 60 min of extraction time. The filtrate was filtered through a 0.22 µm organic phase filter membrane (Millipore, USA) for subsequent HPLC analysis. Three operation replicates were performed^9^.

### LC–MS/MS Instrumentation and methods

Separation and quantification were achieved by using a Nexera XR HPLC from Shimadzu Corporation (Kyoto, Japan) coupled to a QTRAP 5500 mass spectrometer from ABSciex (Foster City, CA, USA) operated at unit resolution for both quadrupoles. Liquid chromatography is used to separate the different components of the analyte and mass spectrometry is used to detect the content of each component. HPLC–MS/MS, the data acquisition and analysis were managed by Analyst software. The column used was an XSelect^®^ HSS T3 (100 mm × 2.1 mm, 3.5 µm), from Waters Corporation (Milford, MA, USA) and was maintained at 40 °C. The mobile phase is formed by a mixture of 0.1% formic acid in water (A) and 0.1% formic acid in acetonitrile (B) and the ratio between A and B at different time points is presented in Table 1^10^. The injection volume was 5 µL. The column was treated with gradient elution according to the following timetable: initial (50% B), 0–1 min (50% B), 1–1.5 min (50% B), 1.5–2 min (50% B), 2–5 min (100% B), 5–10 min (100% B), 10–11 min (50% B), 11–15 min (50% B). The mass spectrometer was operated using multiple reaction monitoring (MRM) in the positive ion mode. The specific parent/product ions information and optimized mass spectrometric parameters are shown in Table 2, including declustering potential (DP), entrance potential (EP), collision energy (CE) and collision cell exit potential (CXP). Curtain gas (20 psi), collision gas (medium), ion source GS1 (27 psi), ion source GS2 (60 psi), ion spray voltage (5500 V), ion source temperature (600 °C) and activated interface heater were identical for analytes (Table 3)^11^.

**Table 1:** The liquid phase gradient parameter.

Time (min)	% B
1	50
1.5	50
2	50
5	100
10	100
11	50
15	50

**Table 2:** MRM parameters for the quantification of CNM, THY and EUG and CA (IS).

Compounds	Tr (min)	Transition		MS parameters (V)			
		Q1 mass (Da)	Q3 mass (Da)	DP	EP	CXP	CE
Cinnamaldehyde	2.58	132.90	55.00	104.07	11.96	8.11	15.09
Thymol	5.45	151.10	91.00	100.83	11.64	10.87	19.17
Eugenol	4.29	165.30	124.00	19.47	3.83	16.40	17.95
Cinnamic acid	2.92	149.00	77.10	153.44	4.22	11.01	33.92

**Table 3:** Ion source parameters.

Parameter	Setting
Scan type	MRM
Ionization mode	ESI+
Curtain gas	20
Collision gas	Medium
Ion source gas 1	27
Ion source gas 2	60
Source voltage	5500
Temperature	600

### Method validation

The method was validated comprehensively following <<Consensus of method development and validation of liquid chromatography–tandem mass spectrometry in clinical laboratories>>^12^

#### Linearity

CNM, THY, EUG and CA standards were prepared and diluted in solvents that was prepared at a 7:3 ratio in acetonitrile/water, v/v. CA as an internal standard. The standard calibration line was generated by injecting three standard solutions at six concentration levels (S1–S6) in three repetitions with the concentration of the internal standard kept constant. A plot of peak area with respect to the corresponding concentration was used to demonstrate linearity. The linear regression equation and correlation coefficient were calculated by weighted (1/x^2^) least-squares linear regression analysis. Linearity was considered to be acceptable when correlation coefficients were 0.99 or better and calibrators had accuracies of 85–115% and precisions within ± 15% RSD (relative standard deviation)^13^.

#### Lower limit of the measuring interval

The LLOQ (signal-to-noise > 10) was the lowest concentration point of calibration curve at which accuracy (relative error, RE) within 20% and precision below 20% can be considered acceptable^13^.

#### Intra- and inter-day precision

The intra-assay precision and accuracy were tested. In this work, three samples of EUG (10, 50, 200 ng/mL), CNM (500, 2000, 8000 ng/mL) and THY (2000, 10,000, 50000 ng/mL) were tested at three different concentrations over a period of 6 days. The variability of determination was expressed as the RSD%, and the accuracy was expressed as the relative error (RE%). Herein, the accuracy data within ± 15% RE from the nominal values and a precision data within ± 15% RSD were acceptable. However, the precision and accuracy data of the LLOQ should be within ± 20%^11^.

#### Carryover

Carryover was evaluated by three independent experiments each consisted of running two extractions in the sequence of low_1_–high–low_2_, where low_2_ is a re-injection of low_1_. A passing test meant that low_1_ is within 20% of low_2_ and that low_2_ is within 3 standard deviations of the low_1_ value. The standard deviation was determined using low_1_ values^14^.

### Colony formation assay

The antibacterial activity of CNM, THY and EUG were also examined by colony formation assay^15^. Aliquots of 50 μL spore suspension (approximately 10^4^ cells/mL) of *S. aureus* and *E. coli* were mixed with 50 μL of different concentrations of CNM, THY and EUG solution. The mixtures were incubated at 27 °C for 4 h, and then were each plated onto 3 agar plates (20 μL/plate). After incubation at 27 °C for 24 h, the resulting bacterial colonies in each plate were counted^15^.

### Antimicrobial synergy assay

The synergistic antimicrobial effect of the combination of CNM, THY and EUG were assessed by the same as colony formation assay. Bacterial cells are grown and aliquoted into new microcentrifuge tube, and then mixed with 50 μL volume of different combinations of CNM, THY and EUG mixture^15^. The plates were incubated at 27 °C. The fractional inhibitory concentration (FIC) index is a quantitative criterion for assessing the synergism of tested antimicrobial agents. The FIC index was calculated based on the following formula:

$${\text{FIC}}_{{\text{A}}} = \left( {{\text{MIC}}_{{\text{A}}} {\text{in combination}}} \right)/\left( {{\text{MIC}}_{{\text{A}}} {\text{alone}}} \right)$$

$${\text{FIC}}_{{\text{B}}} = \left( {{\text{MIC}}_{{\text{B}}} {\text{in combination}}} \right)/\left( {{\text{MIC}}_{{\text{B}}} {\text{alone}}} \right)$$

$${\text{FIC index}} = {\text{FIC}}_{{\text{A}}} + {\text{FIC}}_{{\text{B}}}$$

A FIC index of < 0.5 indicates synergistic activity, > 0.5–1 indicates additive effects, > 1 to < 2 indifference, and ≥ 2 is considered to be antagonism^16^.

### Trace metals analysis

In this study, we chose 1/6 MIC (CNM + THY) to treat the bacteria. Bacteria cells were grown to log phase and resuspended to a cell density of 3 × 10^8^ cells/mL in LB medium. The medium containing the bacteria cells was centrifugated at 10,000 g at room temperature for 5 min, and the pellet collected and washed with sterile PBS. Bacterial samples were ground with a mortar and pestle to disrupt cells. Approximately, 20 mg of crushed sample was weighed out and suspended in 12 mL of 2% nitric acid (trace element grade). Afterwards, the sample was sonicated for 2 h to depolymerize the cells and obtain a homogeneous suspension. The suspension was then centrifuged for 5 min, the supernatant was then separated and was ready for ICP-MS analysis. This is an important step in order to avoid clogging of the sample introduction system^17^.

All ICP-MS experiments in this study were carried out with a ICAP TQ Inductively-Coupled Plasma Mass Spectrometry system (Thermo, USA), using the operating conditions described in Table 4.

**Table 4:** ICP-MS instrument and plasma conditions for operation.

Instrument operating conditions	Mode of operation	
	Ca, Mg, Fe, Cu, Zn, Se, Pb	V, Cr, Mn, Co, Ni, As, Mo, Ag, Cd, Sn, Ba, Au, Hg, Tl
Ar nebulizer gas flow	1.13 l/min	1.17 l/min
Auxilliary gas flow	0.80 l/min	
Cool gas flow	14.0 l/min	
Plasma power	1550 W	
Measurement units	Cps (counts per second)	
Curve type	linear	
Sample units	μg/l	
Replicates	12	
Sweeps	10	
Dwell time	100 ms	
Channels	3	
Spacing (u)	0.1	
He gas flow	0 mL/min	4.5 mL/min
Measurement mode	STD	KED
Internal standard	^103^Rh, ^209^Bi	^103^Rh, ^209^Bi, ^115^In

### Statistical analysis

All experimental data are shown as means ± SEM, and statistically analyzed by SPSS 22.0 (IBM Inc., Chicago, IL, USA). And GraphPad Prism 8.0.2 was applied for graphical plotting and analysis. Statistical differences between concentration points were assessed using unpaired t-test, *p < 0.05; **p < 0.01; ***p < 0.001.

## Results and discussion

### LC–MS/MS Method

The cinnamic acid (CA) was selected as the internal calibration standard (IS) in the established LC–MS/MS method for quantifying CNM, THY and EUG due to two reasons: first, the chromatographic peaks of CA, CNM, THY and EUG were eluted with a good separation, and second, none of four ingredients were found together in chili essential oil.

**Figure 1:**
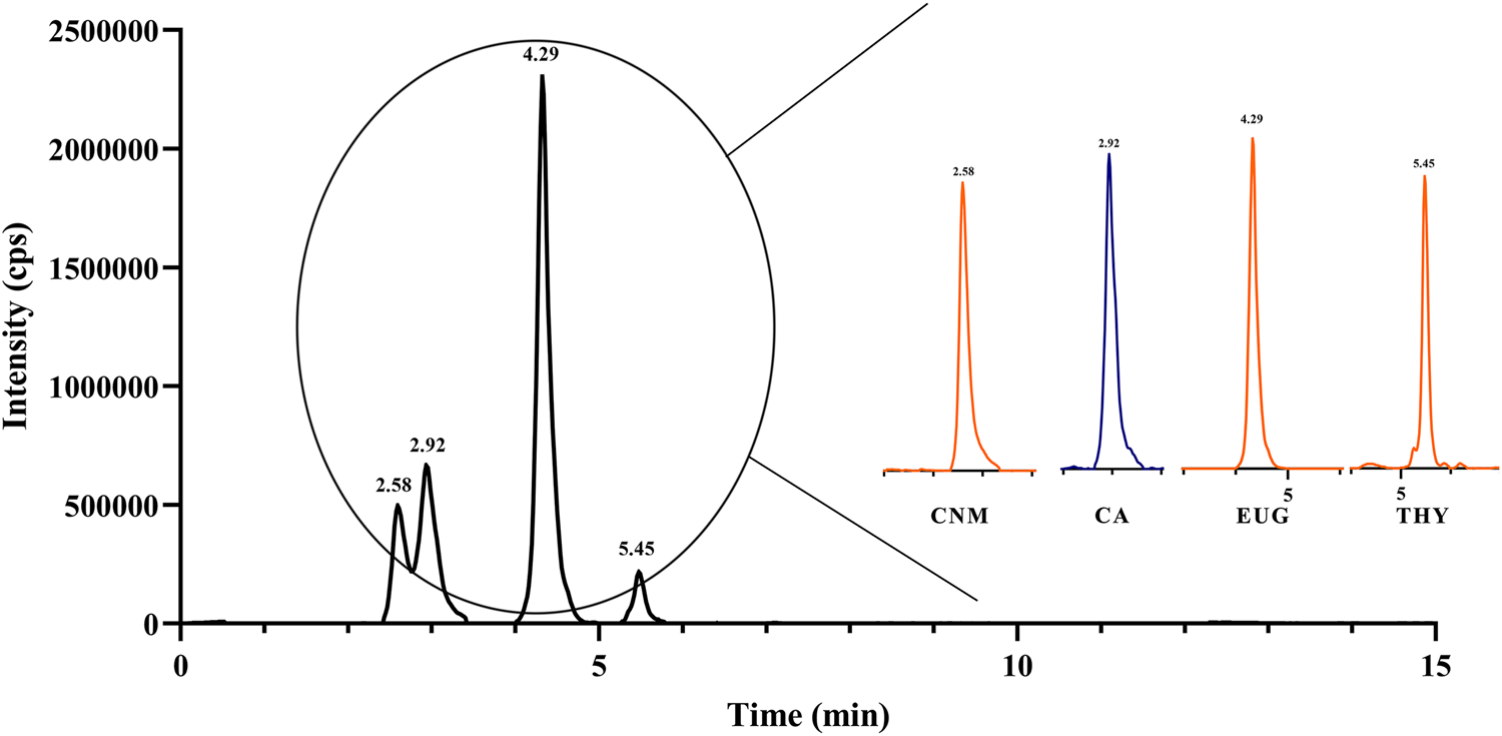
Typical multiple reaction monitoring (MRM) chromatograms of CNM, CA, EUG and THY.

Typical multiple reaction monitoring (MRM) chromatograms of CNM, CA, EUG and THY are displayed in Fig. 1. The concentrations of CNM, EUG and THY in chili essential oil were 1190, 77,700 and 18.4 ng/mL, respectively.

### Method validation

#### Calibration curve and lower limit of quantification (LLOQ)

Calibration curves were prepared using calibration samples. Linear regression analysis was performed by plotting the analyte/IS peak area ratio versus the concentration, and correlation studies were conducted using the Pearson correlation coefficient (r^2^). Table 5 shows the concentration ranges evaluated using 6 calibration points. The calibration curves for three analytes were best fitted with a quadratic regression weighted 1/c^2^. CNM, THY and EUG yielded good linearity over the concentration ranges of 500–10,000 ng/mL, 2000–100,000 ng/mL and 10–400 ng/mL respectively, with mean correlation coefficients (r^2^) of 0.9970, 0.9976 and 0.9957, respectively (Table S1).

**Table 5:** Linear range, correlation coefficient (r^2^) and LLOQ.

Compounds	Regression Equation	r^2^	Linear range (ng/mL)	LLOQ (n = 10)			
				Nominal (ng/mL)	Found (ng/mL)	Accuracy (%)	CV (%)
CNM	y = 0.9735x + 74.878	0.9970	500–10,000	500	490.00	98.00	4.38
THY	y = 0.9108x + 1543.2	0.9976	2000–100,000	2000	2028.30	101.42	3.11
EUG	y = 0.8983x + 7.9582	0.9957	10–400	10	9.61	96.10	2.48

To determine the LLOQ, 10 replicates of samples with set concentrations were measured, the accuracy of results was distributed in the range of 85.30–101.10%, and the CV < 9.73%. The proven LLOQ is lower than the clinical reference interval and fully matches the requirement of medical detection. The lower limit of quantification (LLOQ) of CNM, THY and EUG were determined to be 500, 2000 and 10 ng/mL (Table S2).

#### Intra- and inter-day precision

Precision refers to the proximity of independent measurement results obtained under specified conditions, which reflects the repeatability of detection methods. The intra-day precision was evaluated by analysis of 3 replicates of samples in a single batch, and the inter-day precision was determined by repeating analysis of samples in six independent analytical runs. Intra-day (n = 3) and inter-day (n = 6) precision and accuracy results for low, medium, and high concentration levels are reported in Table 6 and S3. The mean intra-day accuracies ranged from 85.17 to 115.00%, whereas the intra-day precision and inter-day precision, described as the relative standard deviation (RSD) of the calculated concentrations, were below 14.21 and 5.38%, respectively. These results demonstrated the method for quantitative determination of CNM, THY and EUG was reliable and repeatable. The CV% values of intra-, inter-day and overall meet the criteria of method validation, indicating the extreme reproducibility of methods.

**Table 6:** Precision results of CNM, EUG and THY.

Analyte	Concentration (ng/mL)	Day	mean (ng/mL)	Recovery (%)	Intraday (CV)	Interday (CV)
CNM	500	1	461.67	92.33	6.34%	2.33%
		2	429.67	85.93	4.23%	
		3	444.33	88.87	6.90%	
		4	449.67	89.93	6.22%	
		5	448.00	89.60	5.15%	
		6	450.33	90.07	7.90%	
	2000	1	2170.00	108.50	3.23%	4.09%
		2	2053.33	102.67	4.73%	
		3	2003.33	100.17	8.49%	
		4	2116.67	105.83	5.31%	
		5	1930.00	96.50	4.61%	
		6	2053.33	102.67	6.36%	
	8000	1	7026.67	87.83	4.09%	4.72%
		2	7073.33	88.42	3.63%	
		3	7040.00	88.00	3.49%	
		4	7770.00	97.13	0.46%	
		5	7610.00	95.13	5.89%	
		6	7636.67	95.46	0.87%	
THY	2000	1	2076.67	103.83	4.85%	2.94%
		2	1930.00	96.50	9.08%	
		3	2020.00	101.00	1.31%	
		4	1980.00	99.00	8.50%	
		5	1960.00	98.00	6.75%	
		6	2066.67	103.33	10.15%	
	10,000	1	10,966.67	109.67	8.22%	3.14%
		2	11,266.67	112.67	4.00%	
		3	11,500.00	115.00	0.87%	
		4	11,233.33	112.33	2.86%	
		5	10,566.67	105.67	7.71%	
		6	11,466.67	114.67	3.52%	
	50,000	1	48,833.33	97.67	2.48%	1.55%
		2	50,000.00	100.00	1.40%	
		3	50,100.00	100.20	3.60%	
		4	48,666.67	97.33	1.54%	
		5	48,266.67	96.53	5.63%	
		6	48,733.33	97.47	2.21%	
EUG	10	1	9.36	93.57	9.88%	4.21%
		2	9.18	91.80	10.30%	
		3	8.59	85.87	9.34%	
		4	9.52	95.20	10.38%	
		5	9.74	97.37	9.91%	
		6	9.29	92.90	0.49%	
	50	1	49.80	99.60	6.18%	3.72%
		2	53.07	106.13	2.12%	
		3	54.73	109.47	7.22%	
		4	53.73	107.47	6.66%	
		5	50.60	101.20	7.51%	
		6	53.70	107.40	4.55%	
	200	1	170.33	85.17	6.00%	5.38%
		2	193.33	96.67	14.21%	
		3	194.67	97.33	3.26%	
		4	194.33	97.17	5.85%	
		5	195.67	97.83	5.25%	
		6	182.33	91.17	4.43%	

#### Carryover

Table 7 shows after multiple injections of standards and injections of blank samples, no carry-over effect was detected in the negative or positive control chromatograms.

$${\text{Carryover}}\left( \% \right) = \left( {{\text{mean}}_{{{\text{L}} - {\text{L}}}} {-}{\text{mean}}_{{{\text{H}} - {\text{L}}}} } \right)/{\text{mean}}_{{{\text{L}} - {\text{L}}}} *{1}00\%$$

where mean_L–L_ and mean_H–L_ represented the measured concentrations of low-concentration samples injected after the low concentration and the high-concentration samples, respectively. The calculated carryover is within ± 15%, indicating the accuracy of low-concentration samples will not be affected by former injecting.

**Table 7:** Carryover results of CNM, EUG and THY.

Sample	CNM	L–L	H–L	THY	L–L	H–L	EUG	L–L	H–L
L1	1150			6460			23.3		
L2	1210	1210		5980	5980		23.6	23.6	
L3	1170	1170		6440	6440		20.4	20.4	
H1	4340			25,400			96.1		
H2	3810			24,100			94.5		
L4	1120		1120	6530		6530	21.8		21.8
H3	4390			23,600			109		
H4	4310			23,200			109		
L5	1130		1130	6170		6170	23.8		23.8
L6	1040	1040		6420	6420		20.8	20.8	
L7	1090	1090		6760	6760		22.3	22.3	
L8	1110	1110		6490	6490		21.3	21.3	
H5	3880			21,400			89.1		
H6	4120			22,700			103		
H7	3920			22,100			95.6		
H8	4070			23,900			99.7		
L9	1090		1090	6710		6710	18.9		18.9
H10	3780			22,000			94.4		
H11	3890			22,200			93.6		
L10	984		984	6320		6320	19.8		19.8
Mean		1124.00	1081.00		6418.00	6432.50		21.68	21.08
Carryover		− 3.83%			0.23%			− 2.79%	

### Minimal inhibitory concentration (MIC) of CNM, THY and EUG

**Figure 2:**
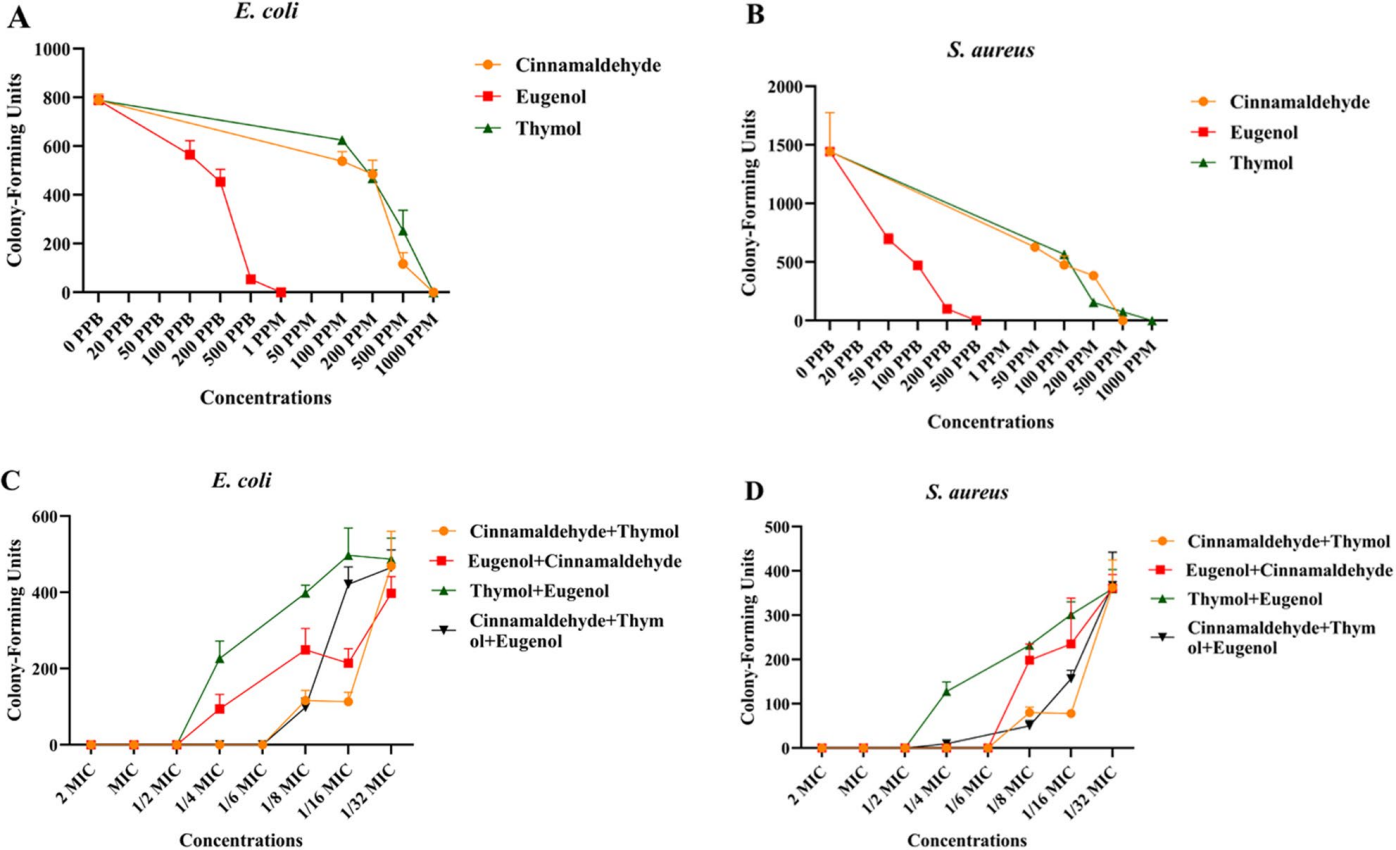
In vitro antibacterial activity of CNM, THY and THY. (**A**) inhibition of each component against *E. coli*; (**B**) inhibition of each component against *S. aureus*; (**C**) Synergistic inhibition of each component on *E. coli*; (**D**) Synergistic inhibition of each component on *S. aureus*.

Colony formation assay demonstrated that CNM, THY and EUG were able to kill *S. aureus* and *E. coli* at all the concentrations tested (Fig. 2a, b and Table S4). The MIC values of CNM killing *S. aureus* and *E. coli* were 1000 and 500 μg/mL, respectively. The MIC values of THY killing *S. aureus* and *E. coli* were 1000 μg/mL. The MIC values of EUG killing *S. aureus* and *E. coli* were 500 ng/mL.

### Synergistic effect of CNM, THY and EUG

Figure 2c and Table 8 shows that a complete synergistic effect was recorded in the case of CNM + THY and CNM + THY + EUG (FICI ≤ 0.5), whereas no synergistic inhibitory effect was observed at low concentrations of CNM + EUG and THY + EUG on *E. coli* (0.5 < FICI < 1). In *E. coli* experiments which exhibited synergism, the mean reduction in the effective drug doses were sixfold for CNM + THY and CNM + THY + EUG. Among the *S. aureus* assays (Fig. 2d), synergism was observed for CNM + THY and CNM + EUG but no synergism was observed for THY + EUG and CNM + THY + EUG. The corresponding mean reductions for the drugs assayed in *S. aureus* strains were sixfold for CNM + THY and CNM + EUG^18^.

**Table 8:** FIC index of each component to *E. coli* and *S. aureus.*

	CNM + THY	CNM + THY	EUG + THY	CNM + EUG + THY
*E. coli*	0.17	0.50	0.50	0.17
*S. aureus*	0.17	0.17	0.50	0.50

### Elemental abundance of microbial cells

In this study, 21 metal elements were measured by ICP-MS in bacteria. Changes in the concentration of metal elements adversely affect bacteria. Membrane stability is attributed to the binding attributed to alkaline earth metals. While the interior of the membrane structure is lipophilic, the inner and outer surfaces contain hydrophilic phosphate molecules^19–23^. Metals stabilize cell membranes and principally, Mg^2+^ and Ca^2+^ are found to neutralize the negative charges on ionized phosphate molecules on the cell membrane surface, and magnesium ions play important roles in stabilizing both tertiary and quaternary structure^24,25^, with low and high ion concentrations impacting the function of ribosomes. Among the *S. aureus* assays, the elevated concentration of Mg, Ca, Fe, Cu, Pb, V, Cr, Mn, Co, As, Ba and Tl was observed in *S. aureus* but Mo and Cd concentrations were lower in cells treated with CNM + THY than control group. Our results revealed changes in intracellular magnesium and calcium ion concentrations in *S. aureus* after treatment, suggesting that we may have affected the cell membrane of the cells.

**Figure 3:**
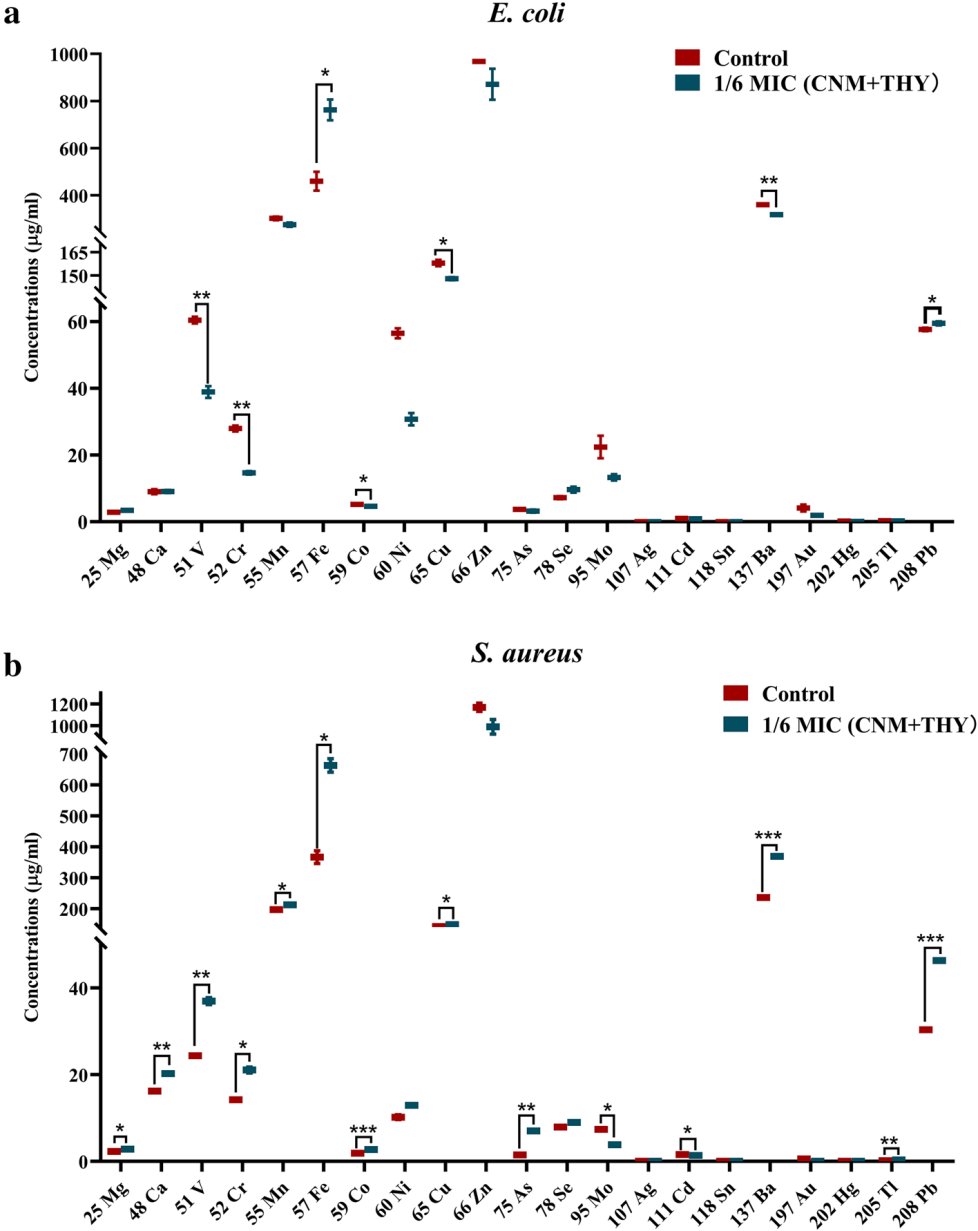
The concentration of trace elements in *E. coli* (**a**) and *S. aureus* (**b**). Statistical differences between concentration points were assessed using unpaired t-test, *p < 0.05; **p < 0.01; ***p < 0.001.

Copper in biological systems presents a formidable problem: it is essential for life, yet highly reactive and a potential source of cell damage. Free intracellular iron would be expected to be low because this metal is bound into specific sites on the proteins and the toxicity of free cationic iron is well known^26–30^, and a marked increase in iron concentration was found in both bacteria after treatment. As shown in Fig. 3 and Table S5, the elevated concentration of Fe was observed in *E. coli* but Cu, V, Cr, Co, Ba and Tl concentrations were lower in cells treated with CNM + THY than control group. Our results suggest that CNM + THY causes damage to bacteria by altering the levels of iron and copper in bacteria.

At higher concentrations, heavy-metal ions form unspecific complex compounds in the cell, which leads to toxic effects^31–34^. Then, the concentration of some elements in the cell changes, resulting in cell damage. Our results suggest that CNM + THY causes changes in cells such as V, Cr, Co, Ba, Tl, Pb, Mn and As.

## Conclusion

We developed a novel LC–MS/MS method for CNM, THY and EUG determination in chili essential oils. CA is used as an IS. The method was demonstrated to be no carryover and the linearity, accuracy and precision of the method for the detection of CNM THY and EUG were found to be in accordance with the specified requirements. Good linearity was reached in the range of 500–10,000 ng/mL, 2000–100,000 ng/mL and 10–400 ng/mL respectively, and the LLOQ of CNM, THY and EUG was determined to be 500, 2000 and 10 ng/mL, respectively. The mean intra-assay accuracy ranged from 85.17 to 115.00%, whereas the intra-assay precision (RSD) was below 14.21%^11^. Then, the results of this study imply that CNM, THY and EUG could be efficiently employed to treat gram-negative and gram-positive infections.

### Supplementary Information

Supplementary Information.

## Data Availability

The datasets generated and/or analysed during the current study are not publicly available due [REASON WHY DATA ARE NOT PUBLIC] but are available from the corresponding author on reasonable request.
