# Supplementary material for: Determination of cinnamaldehyde, thymol and eugenol in essential oils by LC–MS/MS and antibacterial activity of them against bacteria
Source: Sci Rep. 2024 May 30;14:12424. doi: 10.1038/s41598-024-63114-8 (PMC11139912; doi:10.1038/s41598-024-63114-8)
Supplement: Supplementary file 1 — Supplementary Information. [file 41598_2024_63114_MOESM1_ESM.docx]

Determination of cinnamaldehyde, thymol and eugenol in essential oils by LC-MS/MS and antibacterial activity of them against bacteria

Zhi Li^a^, Yan Li^a,b^, Wenbo Cheng^a,b*^

*^a^Tianjin Guoke Medical Engineering and Technology*

*Development Co., Ltd, Tianjin 300300, China*

*^b^ Suzhou Institute of Biomedical Engineering and Technology, Chinese*

*Academy of Sciences, Suzhou 215163, China*

*Correspondence author

Dr. Wenbo Cheng

Suzhou Institute of Biomedical Engineeringand Technology, Chinese Academy of Sciences

Suzhou, 215163, China

E-mail: chengwb@sibet.ac.cn

| **CNM** r^2^=0.9970 |  | **Nominal**  (ng/mL) | **Found** **1** (ng/mL) | **Found** **2** (ng/mL) | **Found** **3** (ng/mL) | **Mean** (ng/mL) |
| --- | --- | --- | --- | --- | --- | --- |
|  | S1 | 500 | 496 | 487 | 501 | 494.67 |
|  | S2 | 1000 | 1000 | 1040 | 983 | 1007.67 |
|  | S3 | 2000 | 2060 | 2090 | 2040 | 2063.33 |
|  | S4 | 4000 | 3910 | 3930 | 4040 | 3960.00 |
|  | S5 | 8000 | 8390 | 8220 | 8060 | 8223.33 |
|  | S6 | 10000 | 9470 | 9340 | 9760 | 9523.33 |

Table S1. Linear measurements of cinnamaldehyde (CNM), thymol (THY), and eugenol (EUG)

| **THY** r^2^=0.9976 |  | **Nominal** (ng/mL) | **Found 1** (ng/mL) | **Found 2** (ng/mL) | **Found 3** (ng/mL) | **Mean** (ng/mL) |
| --- | --- | --- | --- | --- | --- | --- |
|  | S1 | 2000 | 2000 | 1890 | 2090 | 1993.33 |
|  | S2 | 5000 | 4640 | 5560 | 4380 | 4860.00 |
|  | S3 | 10000 | 11000 | 10500 | 9850 | 10450.00 |
|  | S4 | 20000 | 22400 | 21000 | 21900 | 21766.67 |
|  | S5 | 50000 | 48500 | 48500 | 50800 | 49266.67 |
|  | S6 | 100000 | 88300 | 87100 | 98300 | 91233.33 |

| **EUG** r^2^=0.9957 |  | **Nominal** (ng/mL) | **Found 1** (ng/mL) | **Found 2** (ng/mL) | **Found 3** (ng/mL) | **Mean** (ng/mL) |
| --- | --- | --- | --- | --- | --- | --- |
|  | S1 | 10 | 9.59 | 10.2 | 10 | 9.87 |
|  | S2 | 20 | 22 | 17.8 | 21 | 20.10 |
|  | S3 | 50 | 45.7 | 56.7 | 50 | 50.77 |
|  | S4 | 100 | 112 | 113 | 108 | 111.00 |
|  | S5 | 200 | 197 | 181 | 211 | 196.33 |
|  | S6 | 400 | 368 | 369 | 344 | 360.33 |

Table S2. The results of LLOQ test

| LLOQ | | | |
| --- | --- | --- | --- |
|  | **Thymol** | **Cinnamaldehyde** | **Eugenol** |
| 1 | 1970 | 507 | 9.42 |
| 2 | 1990 | 505 | 9.79 |
| 3 | 2090 | 509 | 9.44 |
| 4 | 2030 | 453 | 9.89 |
| 5 | 2010 | 461 | 9.27 |
| 6 | 2140 | 476 | 9.32 |
| 7 | 1970 | 502 | 9.63 |
| 8 | 1948 | 501 | 9.6 |
| 9 | 2040 | 510 | 9.89 |
| 10 | 2095 | 476 | 9.85 |
| Mean(ng/mL) | 2028.3 | 490 | 9.61 |
| Accuracy(%) | 101.42% | 98.00% | 96.10% |
| CV(%) | 3.11% | 4.38% | 2.48% |

Table S3. Inter and Intra Precision results of CNM, EUG and THY

| **Cinnamaldehyde** | | | | | | | | |
| --- | --- | --- | --- | --- | --- | --- | --- | --- |
| 1 | 428 | 415 | 454 | 460 | 456 | 489 |  |  |
| 2 | 476 | 424 | 410 | 471 | 466 | 419 |  |  |
| 3 | 481 | 450 | 469 | 418 | 422 | 443 |  |  |
| **Mean** | 461.67 | 429.67 | 444.33 | 449.67 | 448.00 | 450.33 | **Inter day Mean** | 447.28 |
| **CV** | 6.34% | 4.23% | 6.90% | 6.22% | 5.15% | 7.90% | **CV** | 2.33% |
| 1 | 2250 | 2160 | 2130 | 2020 | 1960 | 2200 |  |  |
| 2 | 2120 | 1970 | 1810 | 2090 | 1830 | 2010 |  |  |
| 3 | 2140 | 2030 | 2070 | 2240 | 2000 | 1950 |  |  |
| **Mean** | 2170.00 | 2053.33 | 2003.33 | 2116.67 | 1930.00 | 2053.33 | **Inter day Mean** | 2054.44 |
| **CV** | 3.23% | 4.73% | 8.49% | 5.31% | 4.61% | 6.36% | **CV** | 4.09% |
| 1 | 7350 | 6920 | 7310 | 7810 | 8100 | 7580 |  |  |
| 2 | 6800 | 6930 | 6830 | 7760 | 7510 | 7620 |  |  |
| 3 | 6930 | 7370 | 6980 | 7740 | 7220 | 7710 |  |  |
| **Mean** | 7026.67 | 7073.33 | 7040.00 | 7770.00 | 7610.00 | 7636.67 | **Inter day Mean** | 7359.44 |
| **CV** | 4.09% | 3.63% | 3.49% | 0.46% | 5.89% | 0.87% | **CV** | 4.72% |

| **Eugenol** | | | | | | | | |
| --- | --- | --- | --- | --- | --- | --- | --- | --- |
| 1 | 9.86 | 8.25 | 8.06 | 10.46 | 9.21 | 9.33 |  |  |
| 2 | 8.29 | 9.15 | 8.19 | 8.49 | 10.85 | 9.24 |  |  |
| 3 | 9.92 | 10.14 | 9.51 | 9.61 | 9.15 | 9.3 |  |  |
| **Mean** | 9.36 | 9.18 | 8.59 | 9.52 | 9.74 | 9.29 | **Inter day Mean** | 9.28 |
| **CV** | 9.88% | 10.30% | 9.34% | 10.38% | 9.91% | 0.49% | **CV** | 4.21% |
| 1 | 47.2 | 52.1 | 58.6 | 49.8 | 47.2 | 52 |  |  |
| 2 | 49 | 54.3 | 54.9 | 54.6 | 49.9 | 56.5 |  |  |
| 3 | 53.2 | 52.8 | 50.7 | 56.8 | 54.7 | 52.6 |  |  |
| **Mean** | 49.80 | 53.07 | 54.73 | 53.73 | 50.60 | 53.70 | **Inter day Mean** | 52.61 |
| **CV** | 6.18% | 2.12% | 7.22% | 6.66% | 7.51% | 4.55% | **CV** | 3.72% |
| 1 | 163 | 179 | 191 | 191 | 207 | 191 |  |  |
| 2 | 182 | 176 | 191 | 185 | 193 | 181 |  |  |
| 3 | 166 | 225 | 202 | 207 | 187 | 175 |  |  |
| **Mean** | 170.33 | 193.33 | 194.67 | 194.33 | 195.67 | 182.33 | **Inter day Mean** | 188.44 |
| **CV** | 6.00% | 14.21% | 3.26% | 5.85% | 5.25% | 4.43% | **CV** | 5.38% |

| **Thymol** | | | | | | | | |
| --- | --- | --- | --- | --- | --- | --- | --- | --- |
| 1 | 2090 | 1920 | 2000 | 1850 | 2110 | 1830 |  |  |
| 2 | 1970 | 1760 | 2050 | 1920 | 1910 | 2140 |  |  |
| 3 | 2170 | 2110 | 2010 | 2170 | 1860 | 2230 |  |  |
| **Mean** | 2076.67 | 1930.00 | 2020.00 | 1980.00 | 1960.00 | 2066.67 | **Inter day Mean** | 2005.56 |
| **CV** | 4.85% | 9.08% | 1.31% | 8.50% | 6.75% | 10.15% | **CV** | 2.94% |
| 1 | 10100 | 11300 | 11500 | 11600 | 11500 | 11400 |  |  |
| 2 | 11900 | 11700 | 11400 | 11100 | 10000 | 11100 |  |  |
| 3 | 10900 | 10800 | 11600 | 11000 | 10200 | 11900 |  |  |
| **Mean** | 10966.67 | 11266.67 | 11500.00 | 11233.33 | 10566.67 | 11466.67 | **Inter day Mean** | 11166.67 |
| **CV** | 8.22% | 4.00% | 0.87% | 2.86% | 7.71% | 3.52% | **CV** | 3.14% |
| 1 | 50200 | 49300 | 50600 | 49100 | 50800 | 49500 |  |  |
| 2 | 47900 | 50000 | 48100 | 47800 | 48600 | 47500 |  |  |
| 3 | 48400 | 50700 | 51600 | 49100 | 45400 | 49200 |  |  |
| **Mean** | 48833.33 | 50000.00 | 50100.00 | 48666.67 | 48266.67 | 48733.33 | **Inter day Mean** | 49100.00 |
| **CV** | 2.48% | 1.40% | 3.60% | 1.54% | 5.63% | 2.21% | **CV** | 1.55% |

Table S4. The colony forming unit of *E. coli* and *S. aureus* in the presence of CNM, THY and EUG.

| ***E. coli*** | | | | |
| --- | --- | --- | --- | --- |
| CNM | 100 ppm | 200 ppm | 500 ppm | 1000 ppm |
| Colony forming unit (mean±SD) | 539±39 | 485±58 | 117±46 | 0 |
| THY | 100ppm | 200 ppm | 500 ppm | 1000 ppm |
| Colony forming unit (mean±SD) | 625±17 | 469±35 | 254±83 | 0 |
| EUG | 100 ppb | 200 ppb | 500 ppb | 1 ppm |
| Colony forming unit (mean±SD) | 565±58 | 454±52 | 543±3 | 0 |

| ***S. aureus*** | | | | |
| --- | --- | --- | --- | --- |
| CNM | 100 ppm | 200 ppm | 500 ppm | 500 ppm |
| Colony forming unit (mean±SD) | 628±12 | 476±76 | 384±36 | 0 |
| THY | 100ppm | 200 ppm | 500 ppm | 1000 ppm |
| Colony forming unit (mean±SD) | 566±16 | 157±39 | 75±17 | 0 |
| EUG | 100 ppb | 200 ppb | 500 ppb | 500 ppb |
| Colony forming unit (mean±SD) | 695±45 | 472±22 | 101±15 | 0 |

Table S5. The concentration of trace elements in *E. coli* and *S. aureus*

| ***E. coli*** | | | | | | |
| --- | --- | --- | --- | --- | --- | --- |
|  | **Control (μg/mL)** | | | **1/6 MIC(CNM+THY) (μg/mL)** | | |
| **25 Mg** | 2.77 | 2.96 | 2.85 | 3.3 | 3.51 | 3.42 |
| **48 Ca** | 8.28 | 9.74 | 9.03 | 8.62 | 9.56 | 8.96 |
| **51 V** | 59.42 | 61.43 | 61.12 | 37.15 | 40.66 | 39.21 |
| **52 Cr** | 27.05 | 28.86 | 27.55 | 14.16 | 15.19 | 14,92 |
| **55 Mn** | 294 | 309 | 303 | 266 | 284 | 277 |
| **57 Fe** | 419 | 499 | 451 | 718 | 806 | 782 |
| **59 Co** | 5.23 | 5.19 | 5.17 | 4.55 | 4.69 | 4.66 |
| **60 Ni** | 55 | 58 | 56 | 32.6 | 28.9 | 31.27 |
| **65 Cu** | 156 | 160 | 161 | 149 | 147 | 149 |
| **66 Zn** | 963 | 973 | 973 | 937 | 805 | 861 |
| **75 As** | 3.65 | 3.77 | 3.69 | 2.72 | 3.67 | 3.21 |
| **78 Se** | 6.76 | 7.67 | 7.14 | 8.73 | 10.53 | 9.69 |
| **95 Mo** | 25.79 | 19.02 | 22.53 | 14.25 | 12.35 | 13.1 |
| **107 Ag** | 0 | 0 | 0 | 0 | 0 | 0 |
| **111 Cd** | 1.05 | 0.98 | 1.02 | 0.87 | 0.86 | 0.86 |
| **118 Sn** | 0 | 0 | 0 | 0 | 0 | 0 |
| **137 Ba** | 358 | 362 | 363 | 317 | 318 | 317 |
| **197 Au** | 5.14 | 3.08 | 4.13 | 2.27 | 1.59 | 1.99 |
| **202 Hg** | 0.21 | 0.15 | 0.18 | 0.11 | 0.1 | 0.13 |
| **205 Tl** | 0.32 | 0.3 | 0.3 | 0.25 | 0.25 | 0.25 |
| **208 Pb** | 57.13 | 58.15 | 57.76 | 58.88 | 60.06 | 59.64 |

| ***S. aureus*** | | | | | | |
| --- | --- | --- | --- | --- | --- | --- |
|  | **Control (μg/mL)** | | | **1/6 MIC(CNM+THY) (μg/mL)** | | |
| **25 Mg** | 2.77 | 2.96 | 2.85 | 3.3 | 3.51 | 3.42 |
| **48 Ca** | 8.28 | 9.74 | 9.03 | 8.62 | 9.56 | 8.96 |
| **51 V** | 59.42 | 61.43 | 61.12 | 37.15 | 40.66 | 39.21 |
| **52 Cr** | 27.05 | 28.86 | 27.55 | 14.16 | 15.19 | 14,92 |
| **55 Mn** | 294 | 309 | 303 | 266 | 284 | 277 |
| **57 Fe** | 419 | 499 | 451 | 718 | 806 | 782 |
| **59 Co** | 5.23 | 5.19 | 5.17 | 4.55 | 4.69 | 4.66 |
| **60 Ni** | 55 | 58 | 56 | 32.6 | 28.9 | 31.27 |
| **65 Cu** | 156 | 160 | 161 | 149 | 147 | 149 |
| **66 Zn** | 963 | 973 | 973 | 937 | 805 | 861 |
| **75 As** | 3.65 | 3.77 | 3.69 | 2.72 | 3.67 | 3.21 |
| **78 Se** | 6.76 | 7.67 | 7.14 | 8.73 | 10.53 | 9.69 |
| **95 Mo** | 25.79 | 19.02 | 22.53 | 14.25 | 12.35 | 13.1 |
| **107 Ag** | 0 | 0 | 0 | 0 | 0 | 0 |
| **111 Cd** | 1.05 | 0.98 | 1.02 | 0.87 | 0.86 | 0.86 |
| **118 Sn** | 0 | 0 | 0 | 0 | 0 | 0 |
| **137 Ba** | 358 | 362 | 363 | 317 | 318 | 317 |
| **197 Au** | 5.14 | 3.08 | 4.13 | 2.27 | 1.59 | 1.99 |
| **202 Hg** | 0.21 | 0.15 | 0.18 | 0.11 | 0.1 | 0.13 |
| **205 Tl** | 0.32 | 0.3 | 0.3 | 0.25 | 0.25 | 0.25 |
| **208 Pb** | 57.13 | 58.15 | 57.76 | 58.88 | 60.06 | 59.64 |
